# Enhanced muscle pump during mild dynamic leg exercise inhibits sympathetic vasomotor outflow

**DOI:** 10.14814/phy2.12070

**Published:** 2014-07-17

**Authors:** Keisho Katayama, Koji Ishida, Mitsuru Saito, Teruhiko Koike, Ai Hirasawa, Shigehiko Ogoh

**Affiliations:** 1 Research Center of Health, Physical Fitness and Sports, Nagoya University, Nagoya, Japan; 2 Faculty of Psychological and Physical Science, Aichigakuin University, Nisshin, Japan; 3 Department of Biomedical Engineering, Toyo University, Kawagoe, Japan

**Keywords:** cardiopulmonary baroreceptors, dynamic leg exercise, sympathetic activity

## Abstract

Muscle sympathetic nerve activity (MSNA) is not increased during leg cycling at light and mild intensities, despite activation of central command and the exercise pressor reflex. We determined whether increasing central blood volume and loading the cardiopulmonary baroreceptors modulate sympathetic vasomotor outflow during leg cycling. To this end, we changed the pedaling frequency to enhance skeletal muscle pump. Subjects performed two leg cycle exercises at differential pedal rates of 60 and 80 rpm (60EX and 80EX trials) for two conditions (with and without MSNA measurement). In each trial, subjects completed leg cycling with a differential workload to maintain constant oxygen consumption (VO_2_). MSNA was recorded via microneurography at the right median nerve of the elbow. Without MSNA measurement, thoracic impedance, stroke volume (SV), and cardiac output (CO) were measured non‐invasively using impedance cardiography. Heart rate and VO_2_ during exercise did not differ between the 60EX and 80EX trials. Changes in thoracic impedance, SV, and CO during the 80EX trial were greater than during the 60EX trial. MSNA during the 60EX trial was unchanged compared with that at rest (25.8 ± 3.1 [rest] to 28.3 ± 3.4 [exercise] bursts/min), whereas a significant decrease in MSNA was observed during the 80EX trial (25.8 ± 2.8 [rest] to 19.7 ± 2.0 [exercise] bursts/min). These results suggest that a muscle pump‐induced increase in central blood volume, and thereby loading of cardiopulmonary baroreceptors, could inhibit sympathetic vasomotor outflow during mild dynamic leg exercise, despite activation of central command and the exercise pressor reflex.

## Abstract (precis)

Muscle sympathetic nerve activity during leg cycling was reduced when central blood volume was manipulated by increasing the pedaling frequency. This result suggest that sympathetic vasomotor outflow is strongly affected by loading of cardiopulmonary baroreceptors at light and mild dynamic leg exercise to maintain arterial blood pressure.

## Introduction

Arterial blood pressure (BP) is regulated via sympathetic vasomotor outflow during dynamic exercise to maintain appropriate blood flow to active muscles. Several neural mechanisms, including the central command and the exercise pressor reflex (mechano‐ and metaboreflex within skeletal muscle), play major roles in this context, affecting the arterial baroreflex (Fadel and Raven 2012; Raven and Chapleau 2014). It is well‐established that activations of these two physiological factors determine sympathetic vasomotor outflow (Saito et al. 1991; Ray and Mark 1993; Ettinger et al. 1996; Raven 2008). Indeed, muscle sympathetic nerve activity (MSNA) increases in proportion to exercise intensity when the exercise is static and performed using small muscle mass (i.e., a static handgrip) (Saito et al. 1986). However, when exercise is dynamic with large muscle mass (i.e., leg cycling), MSNA decreases or does not change from that at rest if the exercise is of light or mild intensity, and gradually rises thereafter in proportion to the increase in workload (Saito et al. 1993; Callister et al. 1994; Ichinose et al. 2008). These results suggest that changes in MSNA are not limited by activations of the central command and the exercise pressor reflex when dynamic leg exercise that is light or mild in intensity is performed.

This interesting phenomenon may be linked to loading of cardiopulmonary baroreceptors, attributable to muscle pump‐induced increases in venous return or central blood volume (Mack et al. 1988; Ray et al. 1993; Ray and Saito 1999; Fadel and Raven 2012; Raven and Chapleau 2014). The cardiopulmonary baroreflex during dynamic exercise has been but seldom studied, and most representative work is performed by Ray et al. (1993). The cited authors showed, for the first time, that MSNA was affected by changes in central blood volume mediated by the cardiopulmonary baroreflex during dynamic exercise in the sitting and supine positions. Unfortunately, it remains unclear whether activation of the central command and the exercise pressor reflex was distinguished from the cardiopulmonary baroreflex in their study, because cardiopulmonary responses and the nature of limb muscle activation may differ when exercise are performed in the sitting and supine positions (Stenberg et al. 1967; Egana et al. 2010). Thus, the effect of the cardiopulmonary baroreflex on the modulation of MSNA remains unclear. Ogoh et al. (2007) showed that enhanced muscle pump‐induced loading of the cardiopulmonary baroreceptors modified carotid‐baroreflex function curve. This suggested that increases in central blood volume associated with the muscle pump might activate the cardiopulmonary baroreceptors and inhibit MSNA (Ray and Saito 1999; Fadel and Raven 2012). Therefore, it was necessary to clarify whether muscle pump‐induced increase in central blood volume, thereby loading the cardiopulmonary baroreceptors, affected sympathetic vasomotor outflow, when central command, exercise pressor reflex, and the posture did not change during exercise.

The purpose of the present study was to clarify whether sympathetic vasomotor activity was suppressed by enhanced muscle pump during dynamic leg exercise. MSNA was recorded from the median nerve by using a microneurographic technique during leg cycling with an increase in central blood volume by increasing pedal frequency (e.g., increasing the frequency of muscle contraction) to enhance the skeletal muscle pump (Gotshall et al. 1996; Ogoh et al. 2007). In addition, we maintained constant oxygen consumption at the differential pedal cadence to keep central command and exercise pressor activation similar (Ogoh et al. 2007). We hypothesized that enhanced muscle pump during mild dynamic leg exercise would inhibit in sympathetic vasomotor outflow.

## Methods

### Ethical approval

The present study was approved by the human research committee of the Research Center of Health, Physical Fitness and Sports at Nagoya University.

### Subjects

Eight healthy males were enrolled in the study; of whom six completed the study (means ± standard error [SE]: age = 23.7 ± 0.6 years, height = 176.0 ± 2.3 cm, body mass = 69.4 ± 3.3 kg). Most subjects participated in moderate‐intensity exercise a couple of times per week, but none were engaged in high‐intensity exercise training. Subjects were informed about the experimental procedures and potential risks involved, and written consent was obtained.

### Measurements

#### Respiratory variables

Minute expired ventilation (VE), tidal volume (VT), breathing frequency (f), oxygen uptake (VO_2_), and carbon dioxide output (VCO_2_) were determined by an online system with a mixing chamber, as in our previous studies (Katayama et al. 2011, 2012, 2013). Subjects breathed through a leak‐free nasal mask (5719; Hans Rudolph, Kansas City, MO). Expired gas volume was measured by a Fleisch pneumotachometer (PN‐230; Arco System, Chiba, Japan). Sample gas was drawn through a sampling tube connected to the pneumotachometer to measure the expired gas fraction. Gas fractions were analyzed by a mass spectrometer (ARCO‐1000; Arco System) that was calibrated and confirmed before each test. Breath‐by‐breath data were measured continuously using customized software on a computer (PC‐9821Ra40; NEC, Tokyo, Japan).

#### Cardiovascular variables

An electrocardiogram (ECG) was measured using a three‐lead electrocardiogram (AB‐621; Nihon Koden, Tokyo, Japan), and HR was calculated from each R‐R interval obtained from the ECG. Thoracic electrical impedance (the ΔZ waveform) and central hemodynamic variables (stroke volume [SV] and cardiac output [CO]) were estimated via non‐invasively using the impedance cardiography (PhysioFlow PF‐05 Lab1; Manatec Biomedical, Paris, France). The ΔZ value was outputted to be positive in this device, and therefore change in the ΔZ was presented negative numbers by convention. Thoracic impedance was used as an index of central blood volume (CBV) (Ebert et al. 1986; Cai et al. 2000). The reliability of SV and CO measurements obtained using this apparatus has been previously confirmed both at rest and during exercise (Charloux et al. 2000). ECG and thoracic impedance signals were sampled at a frequency of 200 Hz using an analog‐to‐digital converter (CBI‐3133B; Interface, Hiroshima, Japan) and stored in a computer (CF‐F8; Panasonic, Osaka, Japan). These variables were averaged over 1‐min both at rest and during exercise. SV was estimated not only by impedance cardiography, but also by beat‐to‐beat measurements of ascending aortic pulse velocity waves. A Doppler echocardiographic technique employing an ultrasound system was used to this end (LOGIQ 5 PRO; GE‐Yokogawa Medical Systems, Tokyo, Japan). The velocity curves were traced offline to obtain average velocity‐time integral (VTI). Aortic cross‐sectional areas were calculated from the aortic diameters (D values) at the sinotubular junction under resting conditions. SV was calculated as follows: SV = *π *× (D / 2)^2^× VTI. CO was the product of SV and HR. These variables were averaged over the last 30 s of every experimental minute. Arterial systolic and diastolic BP (SBP and DBP) were taken from the left arm using an automated BP unit (STBP‐780; Colin Medical Instruments, San Antonio, TX). Automated values determined by the Colin STBP‐780 were confirmed by manual readings taken by the experimenter listening through a headset (Katayama et al. 2011). Arterial BP was monitored at 1‐min intervals throughout the tests. Mean arterial pressure (MBP) was calculated using the following equation: MBP = (SBP − DBP)/3 + DBP.

#### Muscle sympathetic nerve activity

Multiunit muscle sympathetic nerve discharges were recorded by the microneurographic technique using a recording system similar to that in our previous studies (Katayama et al. 2011, 2012, 2013). A tungsten microelectrode with a shaft diameter of 0.1 mm (impedance 1–5 MΩ) was inserted manually by an experimenter into the right median nerve at the cubital fossa. The right arm was fixed using equipment to prevent arm movement artifacts during the leg cycling exercise. After insertion, the electrode was adjusted until MSNA was recorded. Identification of MSNA was based on the following criteria: spontaneous burst discharge synchronized with heartbeat and enhanced by the Valsalva maneuver or breath holding, but showing no change in response to sensory stimuli, such as a loud noise or cutaneous touch (Delius et al. 1972; Vallbo et al. 1979; Fagius and Wallin 1980; Saito et al. 1993). Moreover, subjects were asked to hold their breath to identify MSNA during exercise (at least 5 sec). The neurogram was fed to a differential amplifier and amplified 100,000 times through a band‐pass filter (700–2000 Hz). The neurogram was full‐wave rectified and integrated by a capacitance‐integrated circuit with a time constant of 0.1 sec. The mean voltage neurogram was continuously digitized through an analog‐to‐digital converter with a sampling frequency of 200 Hz and stored electronically. MSNA bursts were identified from the meanvoltage neurogram using a customized computer program‐assisted inspection (Katayama et al. 2011, 2012, 2013), which accounted for the latency from the ECG‐R wave to the sympathetic burst (Fagius and Wallin 1980). MSNA was quantified as burst frequency (BF, bursts/min) and incidence (BI, bursts/100 HR) (Saito et al. 1993; Saito et al., 1997; Katayama et al. 2011, 2012). MSNA burst amplitude and total activity could not be calculated because electromyographic, efferent and afferent nerve activities altered the baseline of the integrated neurogram during dynamic leg cycling in most recordings (Saito et al., 1997; Katayama et al. 2011, 2012, 2013).

### Experimental procedures

All studies were performed at a constant temperature (22–24°C). On day 1, all subjects were familiarized with the equipment to be used in the experiment. Subjects were instructed how to laterally extend both arms and how to hold their arms during leg cycling using an electromechanically braked ergometer in a semirecumbent position (Aerobike 75XL III, Combi, Tokyo, Japan) (Saito et al. 1993; Katayama et al. 2011, 2012, 2013). On day 2, subjects carried out an incremental maximal exercise test using the ergometer. The exercise test began at an initial power output of 90 W, and the workload was increased 15 W per minute until exhaustion (Katayama et al. 2011, 2012, 2013). The pedaling rate was maintained at 60 rpm with the aid of a metronome. VO_2_ and HR were recorded during the test and were averaged every 30 sec afterward. The highest VO_2_ value obtained during the exercise protocol was used as peak VO_2_ (VO_2peak_). Workload at 40% VO_2peak_ was calculated for submaximal exercise tests at a pedal cadence of 60 rpm. On day 3, two preliminary submaximal exercises were performed to determine workload at a pedal cadence of 80 rpm. The pedaling rate was maintained by means of a metronome. Subjects arrived the laboratory and rested for 30 min. Subjects first performed submaximal exercise at 40% VO_2peak_ at a pedaling rate of 60 rpm for 7 min, and we confirmed that VO_2_ attained the steady‐state at a target VO_2_. After 15 min of cessation of the first exercise, subjects performed the second submaximal exercise at a pedal cadence of 80 rpm. The increase in pedal frequency (i.e., muscle contraction frequency) was used to enhance the effect of the muscle pump and increase central blood volume (Gotshall et al. 1996; Ogoh et al. 2007). The second exercise at 80 rpm began at 30% VO_2peak_ at 60 rpm, and the workload at 80 rpm was adjusted to elicit the target VO_2_ that was obtained during the first submaximal exercise at 60 rpm. Thus, the workload at the same VO_2_ was lower (*P* < 0.05) at 80 rpm (58.5 ± 5.4 watts) than at 60 rpm (78.3 ± 4.9 watts), and these workloads were utilized in the submaximal exercise tests on days 4–6. During exercise at pedaling of 60 and 80 rpm, subjects were instructed again how to hold their right arm. On day 4, all subjects performed two submaximal exercises during which we measured MSNA (MSNA test). Subjects arrived at the laboratory and rested for 30 min. Respiratory variables, HR, and MSNA were recorded throughout the experiment. The subjects rested for 3 min. Next, the subjects performed 7 min of exercise and rested for 1 min after cessation of exercise. The procedure was repeated twice, i.e., 60 rpm (control, 60EX trial) or 80 rpm (80EX trial) with a 15‐min interval between trials. The order of exercise at 60 and 80 rpm was randomly assigned and counterbalanced. MSNA recordings during the 60EX and 80EX trials were successful in five of eight subjects. MSNA recording failed in the other three subjects, because the electrodes was displaced from the sympathetic nerve of the muscle or because of signal bursts from electromyographic, efferent, and afferent nerve activities. Such bursts concealed MSNA bursts attributable to arm or body movement. MSNA test was repeated 1 month later in these three subjects, and recording was successful in one. Consequently, six subjects from whom we obtained nerve recordings were used in the analysis. On day 5, two submaximal exercise tests were performed to measure thoracic impedance, SV, and CO (hemodynamic test; i.e., MSNA as not measured) in the six subjects for whom MSNA recordings were available. The procedure was identical to that of the MSNA test. The same procedure was repeated twice, i.e., the 60EX and 80 EX trials, with a 15‐min interval between trials. Subjects were asked to provide their rates of perceived exertion (RPE values) (Borg 1982) during the last minute of exercise. The order of the 60EX and 80EX trials during hemodynamic test of each subject was identical to that during MSNA test. On day 6, five of six subjects performed two submaximal exercises during which SV and CO were measured using the Doppler echocardiographic technique (Doppler test; MSNA measurements were not made). The Doppler technique allowed us to confirm the differences in SV and CO measurements obtained via impedance cardiography during the 60EX and 80EX trials. The order of the two trials performed on day 6 by each subject was identical to that used in MSNA and hemodynamic tests.

### Statistical analysis

Values are expressed as the means ± SE. Respiratory and cardiovascular variables and MSNA values during MSNA and hemodynamic tests were averaged every 1 min throughout the experiment. For all data, the assumption of normal distribution was verified using a Kolmogorov‐Sminov test. Changes in variables during the experiment in each trial were analyzed using a Dunnett test (i.e., vs. the last minute of rest). Comparisons of parameters between the 60EX and 80EX trials were performed using a paired t‐test (parametric test) if the distribution was regular. When the distribution was not regular, the Wilcoxon test (nonparametric test) was used. The SPSS (11.5; SPSS, Tokyo, Japan) statistical package was used only to execute the Kolmogorov‐Sminov test; the StatView (5.0, SAS Institute, Tokyo, Japan) software was utilized for other statistical analyses. A *P* value *P* < 0.05 was considered to indicate statistical significance.

## Results

### Maximal exercise test

Cardiorespiratory parameters and workload at exhaustion during maximal exercise test were as follows: VO_2_ = 3.10± 0.08 l/min, 45.2 ± 2.4 mL/kg/min, HR = 184.7 ± 1.4 beats/min, and workload = 252.5 ± 4.3 watts.

### Submaximal exercise test

#### Baseline descriptive data

There were no significant differences in any of the respiratory variables and HR at rest and during exercise between the MSNA and hemodynamic tests. Thus, we indicate the data for respiratory and cardiovascular variables in the hemodynamic test. Any differences in respiratory and cardiovascular variables of resting baseline were not found between 60EX and 80EX trials (Table 1). There was no significant difference in RPE during exercise between the 60EX and 80EX trials (60EX: 10.0 ± 0.7, 80EX: 10.2 ± 0.6).

**Table 1.** Respiratory, cardiovascular and MSNA variables during experiment

	Trials	Rest	Exercise	Recovery
VE (L/min)	60EX	11.4 ± 1.0	32.9 ± 2.0*	24.0 ± 1.2*
	80EX	11.3 ± 0.4	33.4 ± 1.5^†^	22.5 ± 1.7^†^
VT (L)	60EX	0.65 ± 0.10	1.38 ± 0.08*	1.39 ± 0.11*
	80EX	0.65 ± 0.04	1.39 ± 0.11^†^	1.30 ± 0.10^†^
f (breaths/min)	60EX	15.3 ± 1.4	23.9 ± 1.4*	18.2 ± 1.7*
	80EX	15.6 ± 1.1	24.5 ± 2.1^†^^§^	19.0 ± 1.3^†^
VO_2_ (L/min)	60EX	0.28 ± 0.02	1.22 ± 0.06*	0.82 ± 0.06*
	80EX	0.29 ± 0.02	1.23 ± 0.07^†^	0.79 ± 0.05^†^
VCO_2_ (L/min)	60EX	0.22 ± 0.01	1.14 ± 0.04*	0.74 ± 0.05*
	80EX	0.23 ± 0.02	1.16 ± 0.05^†^	0.74 ± 0.05^†^
SBP (mmHg)	60EX	120.0 ± 3.3	153.2 ± 7.4*	140.5 ± 8.9*
	80EX	120.5 ± 6.2	152.8 ± 8.4^†^	141.3 ± 9.4^†^
DBP (mmHg)	60EX	68.2 ± 4.3	63.5 ± 2.8	71.8 ± 2.2
	80EX	71.7 ± 3.0	64.3 ± 4.2	68.8 ± 2.8
MBP (mmHg)	60EX	84.8 ± 3.5	91.4 ± 4.4*	94.7 ± 2.4*
	80EX	87.7 ± 3.5	90.8 ± 2.4^†^	93.0 ± 4.4
MSNA BI (bursts/100 HR)	60EX	38.7 ± 3.6	28.2 ± 3.1*	39.9 ± 5.9
	80EX	41.3 ± 3.7	18.3 ± 1.8^†^^§^	34.8 ± 5.1

Values are mean ± SE (*n* = 6). Values are averaged over the last minutes of each session. VE, expired minute ventilation; VT, tidal volume; f, breathing frequency; VO_2_, oxygen uptake; VCO_2_, carbon dioxide output; SBP, systolic blood pressure; DBP, diastolic blood pressure; MBP, mean blood pressure; MSNA BI, muscle sympathetic nerve activity bursts incidence; *P < 0.05 vs. at Rest in the 60EX trial. ^†^*P* < 0.05 vs. at Rest in the 80EX trial. ^§^*P* < 0.05 60EX vs. 80EX.

#### Respiratory variables

All respiratory variables were increased significantly during exercise (Table 1). There were no significant differences in VE, VT, VO_2_, or VCO_2_ between the two trials throughout the experiment. The f value of the 80EX trial was slightly but significantly higher than that of the 60EX trial.

#### Cardiovascular variables

**Figure 1.**
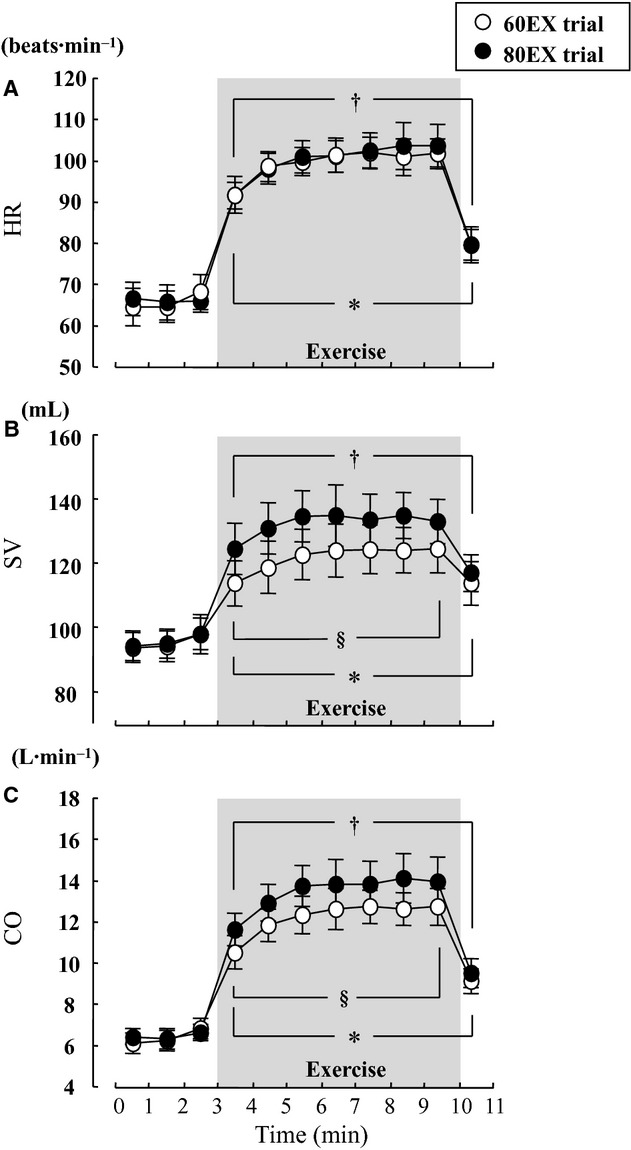
Changes in HR (A), SV (B), and CO (C) during the 60EX and 80EX trials. *P < 0.05 vs. the last minute of rest in the 60EX trial. ^†^*P* < 0.05 vs. the last minute rest in the 80EX trial. ^§^P < 0.05 when comparing the 60EX and 80EX trials.

**Figure 2.**
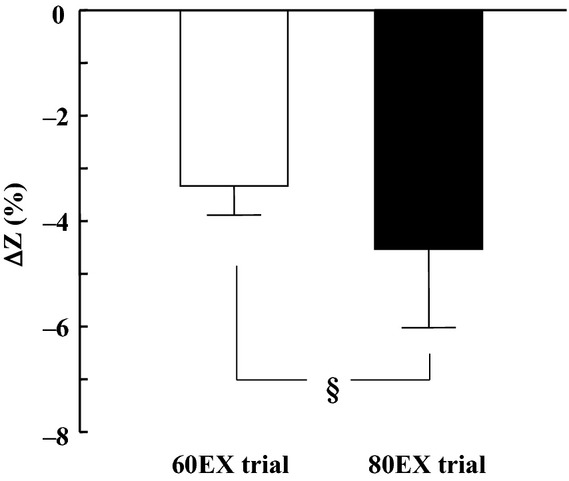
Changes in thoracic impedance ((Z values) from rest to exercise in the 60EX and 80EX trials. ^§^*P* < 0.05 when comparing the 60EX and 80EX trials.

HR increased significantly during exercise in both trials, and this HR response to exercise was not different between the 60EX and 80EX trials (Fig. 1A). SBP and MBP increased, while DBP was unchanged during exercise (Table 1). There were no significant differences in BP variables between trials. Thoracic impedance (ΔZ) changed (from the resting values) in both exercise trials, and the change during the 80EX trial was greater (*P* < 0.05) than that during the 60EX trial (Fig. 2). The SV and CO levels (measured by impedance cardiography) during the 80EX trial were significantly higher than those of the 60EX trial (Fig. 1B and C). Similarly, the SV and CO values obtained via Doppler echocardiography were higher (*P* < 0.05) during the 80EX trial than those in the 60EX trial (Table 2).

**Table 2.** Cardiac variables in the Doppler test

	Trials	Rest	Exercise	Recovery
HR (beats/min)	60EX	70.0 ± 2.8	104.5 ± 4.1^*^	78.0 ± 4.2^*^
	80EX	71.2 ± 1.4	105.5 ± 3.1^†^	79.0 ± 3.7^†^
SV (mL)	60EX	83.0 ± 4.1	106.6 ± 6.9^*^	100.2 ± 5.9^*^
	80EX	81.5 ± 4.6	114.6 ± 7.4^†^^§^	100.8 ± 3.9^†^
CO (L/min)	60EX	5.9 ± 0.2	11.5 ± 0.6^*^	7.7 ± 0.3^*^
	80EX	5.8 ± 0.3	12.3 ± 0.6^†^^§^	7.9 ± 0.3^†^

Values are mean ± SE (*n* = 5). Values are averaged over the last 30 sec of each session. HR, heart rate; SV, stroke volume; CO, cardiac output. SV and CO were obtained by the Doppler echocardiography. *P < 0.05 vs. at Rest in the 60EX trial. ^†^*P* < 0.05 vs. at Rest in the 80EX trial. ^§^*P* < 0.05 60EX trial vs. 80EX trial.

#### Muscle sympathetic nerve activity

**Figure 3.**
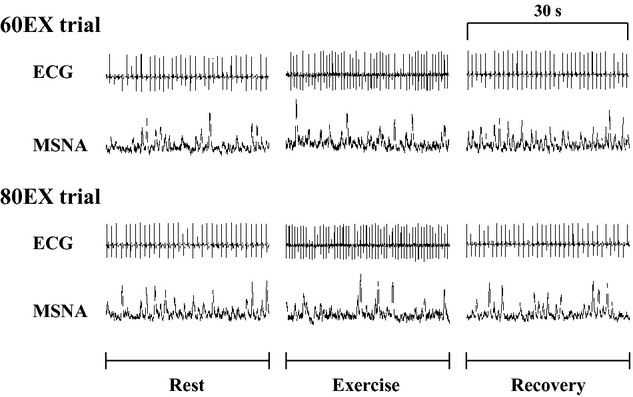
Representative ECG and MSNA recordings made during the 60EX and 80 EX trials.

**Figure 4.**
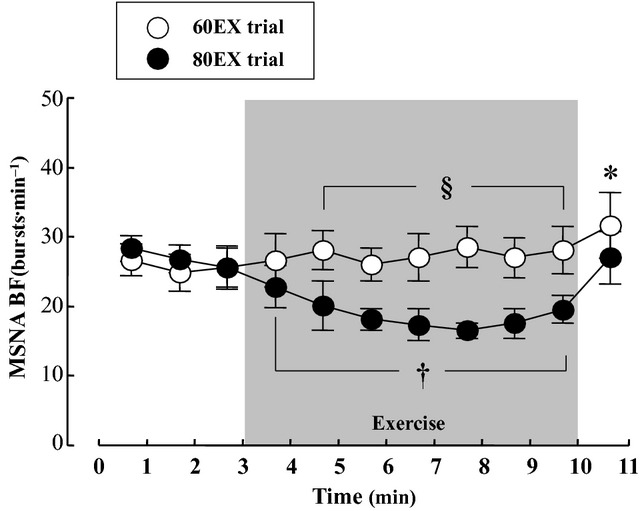
Changes in MSNA BF in the 60EX and 80 EX trials. *P < 0.05 vs. the last minute of rest in the 60EX trial. ^†^*P* < 0.05 vs. the last minute of rest in the 80EX trial. ^§^*P* < 0.05 when comparing the 60EX and 80EX trials.

Representative MSNA recordings and changes in MSNA BF are presented in Fig. 3. MSNA BF did not change during exercise in the 60EX trial, while MSNA BF decreased significantly during exercise in the 80EX trial (Fig. 4). MSNA BF during exercise in the 80EX trial was lower (*P* < 0.05) than that in the 60EX trial. MSNA BI decreased significantly during exercise in both trials (Table 1), and MSNA BI during exercise in the 80EX trial was lower (*P* < 0.05) than that in the 60EX trial.

## Discussion

The major finding of the present study was that MSNA BF fell during leg cycling at mild intensity when the pedaling frequency was increased (thus enhancing muscle pump). This result supports the hypothesis that no change or a decrease in sympathetic vasomotor outflow during dynamic leg exercise up to mild intensity may be due to muscle pump‐induced increase in central blood volume, thereby loading the cardiopulmonary baroreceptors. The results from this study provide additional information concerning the mechanism of the MSNA response to dynamic leg exercise performed at mild intensity.

### Effect of the muscle pump‐induced increase in central blood volume on MSNA

Several previous studies have attempted to evaluate changes in MSNA during dynamic leg exercise, and revealed that MSNA tended to decrease or was unchanged at light and mild exercise intensities compared with that at rest (Seals et al. 1988; Saito and Mano 1991; Ray et al. 1993; Saito et al. 1993; Callister et al. 1994; Ichinose et al. 2008; Katayama et al. 2011). Similarly, in the present study, MSNA BF during exercise in the 60EX trial showed little change from resting values, as shown in Fig. 4. Also, MSNA BI decreased during exercise in the 60EX trial (Table 1). One possible explanation for this observation is that dynamic leg exercise‐induced muscle pump was accompanied by an increase in central blood volume. Ray et al. (1993) compared MSNA responses to dynamic a one‐legged knee exercise performed in the sitting and supine positions. They compared MSNA response to dynamic exercise between supine and upright positions and noted that changes in central blood volume affected the MSNA response to dynamic exercise. In addition, Vollianitis and Secher (Volianitis and Secher 2002) reported that arterial BP was reduced to a level below that achievable upon arm exercise alone when leg‐cycling was added to an arm‐cranking exercise. These results suggest that leg muscle pump strongly affects BP rather than the central command or the exercise pressor reflex. In addition, changing central blood volume affected peripheral vascular resistance (Donald and Shepherd 1978), and the extent of CO is negatively correlated with MSNA (Charkoudian et al. 2005). From these findings, it would be expected that loading of cardiopulmonary baroreceptors by a muscle pump‐induced increase in central blood volume would suppress sympathetic vasomotor activity during dynamic leg exercise (Ray et al. 1993; Ray and Saito 1999; Fadel and Raven 2012; Raven and Chapleau 2014).

In the present study, we utilized a traditional method (an increase in pedal frequency from 60 to 80 rpm) to further enhance skeletal muscle pump frequency (Gotshall et al. 1996; Rowland and Lisowski 2001; Ogoh et al. 2007). This manipulated central blood volume in a controlled manner; SV and CO increased significantly during leg cycling at 80 rpm (the 80EX trial) compared with the values recorded at 60 rpm (the 60EX trial) (Fig. 1). Since CO is equal to venous return under steady‐state conditions (Badeer 1981; Young 2010), increased CO during the 80EX trial indicates enhanced central blood volume. Moreover, thoracic impedance, an index of central blood volume, was higher during the 80EX trial than the 60EX trial (Fig. 2). Consequently, MSNA BF decreased during the 80EX trial (Fig. 4), and MSNA BI (Table 1) during this trial was lower than during the 60EX trial, suggesting that enhanced muscle pump, which caused loading of cardiopulmonary baroreceptors, could inhibit sympathetic vasomotor outflow during dynamic leg exercise at mild intensity.

In an animal study, the BP response at the onset of exercise was not maintained when the cardiopulmonary receptors operated alone (without arterial baroreflex) (Walgenbach and Donald 1983). They concluded that cardiopulmonary receptors do not have a significant role in BP regulation. However, Ogoh et al. (2007) reported that loading of cardiopulmonary baroreceptors modified the arterial baroreflex evident during steady‐state exercise. In addition, orthostatic stress increased peripheral vascular resistance via unloading of cardiopulmonary baroreceptors to maintain the required BP during mild‐intensity exercise (Mack et al. 1988). These earlier findings indicated that the cardiopulmonary and arterial baroreflexes interacted to regulate BP during exercise. Similarly, in the present study, sympathetic vasoconstriction outflow was suppressed by loading of cardiopulmonary baroreceptors during the 80EX trial. Interestingly, BP during exercise in the 80EX trial did not differ from that in the 60EX trial (Table 1). Therefore, suppression of sympathetic vasoconstriction outflow by the cardiopulmonary reflex in the 80EX trial may prevent overshooting of BP during exercise. Taking these observations into consideration, cardiopulmonary receptors could play an important role for sympathetic vasomotor outflow to maintain adequate BP against changes in central blood volume during steady‐state mild dynamic exercise.

We need to consider other possible mechanisms affecting MSNA during dynamic leg exercise. First, it is necessary to consider energy expenditure which is related to central command and the exercise pressor reflex. It is well known that an increase in pedaling cadence induces a rise in gross energy expenditure at a constant load because internal work is altered (Lollgen et al. 1980; Wells et al. 1986). Thus, increases in cycle pedaling cadence may produce greater activation of central command and the exercise pressor reflex when workload is kept constant. In order to apply the same central command and exercise pressor reflex, we used differential workloads to obtain the same energy expenditure (i.e., VO_2_) during the 60EX and 80EX trials (Table 1) (Ogoh et al. 2007). Similar to VO_2_, there were no significant differences in HR and RPE between the two trials. Therefore, we suppose that central command inputs were similar during two trials types; although, it is difficult to estimate the extent thereof.

In terms of the exercise pressor reflex, the metaboreflex and mechanoreflex within skeletal muscle contribute to changes in MSNA during exercise. In an animal study, it has been revealed that group IV muscle afferent was increased during low intensity dynamic exercise (Adreani et al. 1997). An increase in muscle contraction frequency may limit muscle blood flow, thereby triggering metabolite accumulation. However, Ferreira et al. (2006) found no difference in the extent of vastus lateralis deoxygenation (assessed using near–infrared spectroscopy) during cycling at 60 and 100 rpm, indicating that the increase in contraction frequency did not impair blood flow to the muscle. Further, the VCO_2_ value obtained during the 80EX trial did not differ from those of the 60EX trial (Table 1). Collectively, although metaboreflex during dynamic leg cycling at low intensity could enhance MSNA, it seems likely that the extent of metaboreflex did not differ between the 60EX and 80EX trials. As for the mechanoreflex, mechanoreceptor loading certainly increases as muscle contraction frequency rises, and group III muscle afferent was enhanced during dynamic exercise at low intensity (Adreani et al. 1997). Passive stretching of the hindlimb muscle significantly increased renal sympathetic nerve activity (Matsukawa et al. 1990). In addition, MSNA has been reported to increase during passive muscle stretching in humans (Cui et al. 2006). These findings suggest that an increase in mechanoreceptor loading during the 80EX trial could enhance, not inhibit, MSNA.

Finally, respiratory modulation of MSNA should be mentioned (Hagbarth and Vallbo 1968; Eckberg et al. 1985; Dempsey et al. 2002). In the present study, subjects adopted a high breathing frequency during the 80EX trial; however, this difference was small. Seals et al. (1990) showed that differences in depths and patterns of breathing influenced the within‐breath MSNA modulation. However, St Croix et al. (2000) found that MSNA was unchanged when the breathing frequency increased threefold (via voluntary hyperpnea). Therefore, it is unlikely that the slight difference in breathing frequency between the 60EX and 80EX trials significantly affected MSNA; although, we cannot entirely role out respiratory modulation of MSNA.

### Technical considerations and limitations

Several technical considerations and limitations of the study should be noted. One limitation was the use of thoracic electrical impedance as an index of central blood volume; we could not obtain direct measurements of central venous pressure. Previous studies found that changes in thoracic impedance relates to central venous pressure during lower body positive or negative pressure (Ebert et al. 1986; Cai et al. 2000). As in the present study, Ogoh et al. (2007) found that thoracic impedance decreased when the pedal cadence rose from 60 to 80 rpm (at the same VO_2_) during submaximal leg cycling at mild intensity. In addition to thoracic impedance, we measured the central hemodynamic variables SV and CO by both impedance cardiography and a Doppler echocardiographic technique. As CO is equal to the venous return under steady‐state conditions (Badeer 1981; Young 2010), we presumed that changes in central blood volume were reflected in the CO values. Although the absolute values of SV and CO obtained by the Doppler technique were lower than those derived by the impedance method (in line with data of a previous study) (Christie et al. 1987), both SV and CO were significantly higher during the 80EX trial (Fig. 1 and Table 2). Taking these observations into consideration, it seems reasonable to suggest that central blood volume increased during the 80EX trial of the present study.

It is necessary to consider exercise intensity and duration. We utilized mild exercise intensity (40% VO_2peak_ at 60 rpm), because the percentage of successful MSNA recordings is high when the exercise intensity is mild and movements of the arm and the body become greater during leg cycling with a high pedaling rate (Katayama et al. 2012, 2013). The extent of contribution of cardiopulmonary baroreceptor to sympathetic vasoconstriction outflow would alter with increasing exercise intensity, as well as other mechanisms (e.g., metaboreflex) (Ray et al. 1993; Fisher et al. 2005; Boushel 2010). As for the exercise duration, Saito et al. (1997) and Ray et al. (1993) demonstrated a progressive increase in MSNA during 30‐ and 40‐min of dynamic leg exercise at mild intensity. Therefore, it may be that the influence of the cardiopulmonary receptors overwhelms when exercise intensity is high and exercise duration prolonged (Ray et al. 1993).

Another limitation of this study was the limited number and characteristics of subjects. Clinically, cardiopulmonary baroreceptor is particularly important to regulate arterial BP at rest and during exercise. It has been reported that resting MSNA in obesity and patients with heart failure and hypertension is higher than in healthy individuals (Yamada et al. 1989; Grassi et al. 2003; Witte et al. 2008). Furthermore, cardiopulmonary reflex is impaired in patients with heart failure (Dibner‐Dunlap et al. 1996). Thus, it is assumed that the MSNA response to changes in central hemodynamics during dynamic leg exercise in patients differs from that in healthy subjects.

## Conclusion

MSNA BF during leg cycling at mild intensity was reduced when central blood volume was increased by elevating pedaling frequency (e.g., muscle contraction frequency). These results suggest that a muscle pump‐induced increase in central blood volume, and thereby loading of cardiopulmonary baroreceptors, could inhibit sympathetic vasomotor outflow during mild dynamic leg exercise, despite activation of central command and the exercise pressor reflex.

## Conflict of Interest

No conflicts of interest, financial or otherwise, are declared by the authors.

## Acknowledgements

We thank Dr. E. Iwamoto (Sapporo Medical University), Professor Y. Yasuda (Toyohashi University of Technology), Dr. R. Kime, Dr. N. Murase, and Miss S. Fuse (Tokyo Medical University) for their assistance during the study.

## References

[b1] Adreani C. M. Hill J. M. Kaufman M. P. 1997 Responses of group III and IV muscle afferents to dynamic exercise. J. Appl. Physiol.; 82:1811-1817. 9173945 10.1152/jappl.1997.82.6.1811

[b2] Badeer H. S. 1981 Cardiac output and venous return as interdependent and independent variables. Cardiology; 67:65-72. 6974044 10.1159/000173230

[b3] Borg G. A. V. 1982 Psychophysical bases of perceived exertion. Med. Sci. Sports Exerc.; 14:377-381. 7154893

[b4] Boushel R. 2010 Muscle metaboreflex control of the circulation during exercise. Acta Physiol. (Oxf.); 199:367-383. 20353495 10.1111/j.1748-1716.2010.02133.x

[b5] Cai Y. Holm S. Jenstrup M. Stromstad M. Eigtved A. Warberg J. 2000 Electrical admittance for filling of the heart during lower body negative pressure in humans. J. Appl. Physiol.; 89:1569-1576. 11007597 10.1152/jappl.2000.89.4.1569

[b6] Callister R. Ng A. V. Seals D. R. 1994 Arm muscle sympathetic nerve activity during preparation for and initiation of leg‐cycling exercise in humans. J. Appl. Physiol.; 77:1403-1410. 7836146 10.1152/jappl.1994.77.3.1403

[b7] Charkoudian N. Joyner M. J. Johnson C. P. Eisenach J. H. Dietz N. M. Wallin B. G. 2005 Balance between cardiac output and sympathetic nerve activity in resting humans: role in arterial pressure regulation. J. Physiol.; 568:315-321. 16037092 10.1113/jphysiol.2005.090076 PMC1474766

[b8] Charloux A. Lonsdorfer‐Wolf E. Richard R. Lampert E. Oswald‐Mammosser M. Mettauer B. 2000 A new impedance cardiograph device for the non‐invasive evaluation of cardiac output at rest and during exercise: comparison with the “direct” Fick method. Eur. J. Appl. Physiol.; 82:313-320. 10958374 10.1007/s004210000226

[b9] Christie J. Sheldahl L. M. Tristani F. E. Sagar K. B. Ptacin M. J. Wann S. 1987 Determination of stroke volume and cardiac output during exercise: comparison of two‐dimensional and Doppler echocardiography, Fick oximetry, and thermodilution. Circulation; 76:539-547. 3621519 10.1161/01.cir.76.3.539

[b10] Cui J. Blaha C. Moradkhan R. Gray K. S. Sinoway L. I. 2006 Muscle sympathetic nerve activity responses to dynamic passive muscle stretch in humans. J. Physiol.; 576:625-634. 16873399 10.1113/jphysiol.2006.116640 PMC1890351

[b11] Delius W. Hagbarth K. E. Hongell A. Wallin B. G. 1972 General characteristics of sympathetic activity in human muscle nerves. Acta Physiol. Scand.; 84:65-81. 5029385 10.1111/j.1748-1716.1972.tb05158.x

[b12] Dempsey J. A. Sheel A. W. St Croix C. M. Morgan B. J. 2002 Respiratory influences on sympathetic vasomotor outflow in humans. Respir. Physiol. Neurobiol.; 130:3-20. 12380012 10.1016/s0034-5687(01)00327-9

[b13] Dibner‐Dunlap M. E. Smith M. L. Kinugawa T. Thames M. D. 1996 Enalaprilat augments arterial and cardiopulmonary baroreflex control of sympathetic nerve activity in patients with heart failure. J. Am. Coll. Cardiol.; 27:358-364. 8557906 10.1016/0735-1097(95)00484-x

[b14] Donald D. E. Shepherd J. T. 1978 Reflexes from the heart and lungs: physiological curiosities or important regulatory mechanisms. Cardiovasc. Res.; 12:446-469. 363264

[b15] Ebert T. J. Smith J. J. Barney J. A. Merrill D. C. Smith G. K. 1986 The use of thoracic impedance for determining thoracic blood volume changes in man. Aviat. Space Environ. Med.; 57:49-53. 3942570

[b16] Eckberg D. L. Nerhed C. Wallin B. G. 1985 Respiratory modulation of muscle sympathetic and vagal gardiac outflow in man. J. Physiol.; 365:181-196. 4032310 10.1113/jphysiol.1985.sp015766 PMC1192996

[b17] Egana M. Ryan K. Warmington S. A. Green S. 2010 Effect of body tilt angle on fatigue and EMG activities in lower limbs during cycling. Eur. J. Appl. Physiol.; 108:649-656. 19890660 10.1007/s00421-009-1254-8

[b18] Ettinger S. M. Silber D. H. Collins B. G. Gray K. S. Sutliff G. Whisler S. K. 1996 Influences of gender on sympathetic nerve responses to static exercise. J. Appl. Physiol.; 80:245-251. 8847310 10.1152/jappl.1996.80.1.245

[b19] Fadel P. J. Raven P. B. 2012 Human investigations into the arterial and cardiopulmonary baroreflexes during exercise. Exp. Physiol.; 97:39-50. 22002871 10.1113/expphysiol.2011.057554 PMC3253263

[b20] Fagius J. Wallin B. G. 1980 Sympathetic reflex latencies and conduction velocities in normal man. J. Neurol. Sci.; 47:433-448. 7420119 10.1016/0022-510x(80)90098-2

[b21] Ferreira L. F. Lutjemeier B. J. Townsend D. K. Barstow T. J. 2006 Effects of pedal frequency on estimated muscle microvascular O2 extraction. Eur. J. Appl. Physiol.; 96:558-563. 16369819 10.1007/s00421-005-0107-3

[b22] Fisher J. P. Bell M. P. White M. J. 2005 Cardiovascular responses to human calf muscle stretch during varying levels of muscle metaboreflex activation. Exp. Physiol.; 90:773-781. 16049058 10.1113/expphysiol.2005.030577

[b23] Gotshall R. W. Bauer T. A. Fahrner S. L. 1996 Cycling cadence alters exercise hemodynamics. Int. J. Sports Med.; 17:17-21. 8775571 10.1055/s-2007-972802

[b24] Grassi G. Seravalle G. Quarti‐Trevano F. Dell'Oro R. Bolla G. Mancia G. 2003 Effects of hypertension and obesity on the sympathetic activation of heart failure patients. Hypertension; 42:873-877. 14568999 10.1161/01.HYP.0000098660.26184.63

[b25] Hagbarth K. E. Vallbo A. B. 1968 Pulse and respiratory grouping of sympathetic impulses in human muscle‐nerves. Acta Physiol. Scand.; 74:96-108. 4235387 10.1111/j.1748-1716.1968.tb04218.x

[b26] Ichinose M. Saito M. Fujii N. Ogawa T. Hayashi K. Kondo N. 2008 Moduration of the control of muscle sympathetic nerve activity during incremental leg cycling. J. Physiol.; 586:2753-2766. 18403425 10.1113/jphysiol.2007.150060 PMC2536590

[b27] Katayama K. Ishida K. Iwamoto E. Iemitsu M. Koike T. Saito M. 2011 Hypoxia augments muscle sympathetic neural response to leg cycling. Am. J. Physiol. Regul. Integr. Comp. Physiol.; 301:R456-R464. 21593431 10.1152/ajpregu.00119.2011

[b28] Katayama K. Iwamoto E. Ishida K. Koike T. Saito M. 2012 Inspiratory muscle fatigue increases sympathetic vasomotor outflow and blood pressure during submaximal exercise. Am. J. Physiol. Regul. Integr. Comp. Physiol.; 302:R1167-R1175. 22461178 10.1152/ajpregu.00006.2012

[b29] Katayama K. Yamashita S. Ishida K. Iwamoto E. Koike T. Saito M. 2013 Hypoxic effects on sympathetic vasomotor outflow and blood pressure during exercise with inspiratory resistance. Am. J. Physiol. Regul. Integr. Comp. Physiol.; 304:R374-R382. 23283938 10.1152/ajpregu.00489.2012

[b30] Lollgen H. Graham T. Sjogaard G. 1980 Muscle metabolites, force, and perceived exertion bicycling at varying pedal rates. Med. Sci. Sports Exerc.; 12:345-351. 7453512

[b31] Mack G. Nose H. Nadel E. R. 1988 Role of cardiopulmonary baroreflexes during dynamic exercise. J. Appl. Physiol.; 65:1827-1832. 3182543 10.1152/jappl.1988.65.4.1827

[b32] Matsukawa K. Wall P. T. Wilson L. B. Mitchell J. H. 1990 Reflex responses of renal nerve activity during isometric muscle contraction in cats. Am. J. Physiol.; 259:H1380-H1388. 2240239 10.1152/ajpheart.1990.259.5.H1380

[b33] Ogoh S. Fisher J. P. Fadel P. J. Raven P. B. 2007 Increases in central blood volume modulate carotid baroreflex resetting during dynamic exercise in humans. J. Physiol.; 581:405-418. 17317751 10.1113/jphysiol.2006.125112 PMC2075218

[b34] Raven P. B. 2008 Recent advances in baroreflex control of blood pressure during exercise in humans: an overview. Med. Sci. Sports Exerc.; 40:2033-2036. 19018210 10.1249/MSS.0b013e318180bc41

[b35] Raven P. B. Chapleau M. W. 2014 Blood pressure regulation XI: overview and future research directions. Eur. J. Appl. Physiol.; 114:579-586. 24463603 10.1007/s00421-014-2823-z PMC3955090

[b36] Ray C. A. 1993 Muscle sympathetic nerve responses to prolonged one‐legged exercise. J. Appl. Physiol. (1985); 74:1719-1722. 8514687 10.1152/jappl.1993.74.4.1719

[b37] Ray C. A. Mark A. L. 1993 Augmentation of muscle sympathetic nerve activity during fatiguing isometric leg exercise. J. Appl. Physiol.; 75:228-232. 8376268 10.1152/jappl.1993.75.1.228

[b38] Ray C. A. Saito M. 1999 43-51 *in*In: Saltin B. Boushel R. Secher N. H. Mitchell J. H. (eds.). The cardiopulmonary baroreflex. Exercise and circulation in health and disease Champaign Human Kinetics

[b39] Ray C. A. Rea R. F. Clary M. P. Mark A. L. 1993 Muscle sympathetic nerve responses to dynamic one‐legged exercise: effect of body posture. Am. J. Physiol.; 264:H1-H7. 8430836 10.1152/ajpheart.1993.264.1.H1

[b40] Rowland T. Lisowski R. 2001 Hemodynamic responses to increasing cycle cadence in 11‐year old boys: role of the skeletal muscle pump. Int. J. Sports Med.; 22:405-409. 11531031 10.1055/s-2001-16245

[b41] Saito M. Mano T. 1991 Exercise mode affects muscle sympathetic nerve responsiveness. Jpn. J. Physiol.; 41:143-151. 1857017 10.2170/jjphysiol.41.143

[b42] Saito M. Mano T. Abe H. Iwase S. 1986 Responses in muscle sympathetic nerve activity to sustained hand‐grips of different tensions in humans. Eur. J. Appl. Physiol. Occup. Physiol.; 55:493-498. 3769906 10.1007/BF00421643

[b43] Saito M. Abe H. Iwase S. Koga K. Mano T. 1991 Muscle sympathetic nerve responsiveness to static contraction is not altered under hypoxia. Jpn. J. Physiol.; 41:775-783. 1803060 10.2170/jjphysiol.41.775

[b44] Saito M. Tsukanaka A. Yanagihara D. Mano T. 1993 Muscle sympathetic nerve responses to graded leg cycling. J. Appl. Physiol.; 75:663-667. 8226466 10.1152/jappl.1993.75.2.663

[b45] Saito M. Sone R. Ikeda M. Mano T. 1997 Sympathetic outflow to the skeletal muscle in humans increases during prolonged light exercise. J. Appl. Physiol.; 82:1237-1243. 9104861 10.1152/jappl.1997.82.4.1237

[b46] Seals D. R. Victor R. G. Mark A. L. 1988 Plasma norepinephrine and muscle sympathetic discharge during rhythmic exercise in humans. J. Appl. Physiol.; 65:940-944. 3170440 10.1152/jappl.1988.65.2.940

[b47] Seals D. R. Suwarno N. O. Dempsey J. A. 1990 Influence of lung volume on sympathetic nerve discharge in normal humans. Circ. Res.; 67:130-141. 2364488 10.1161/01.res.67.1.130

[b48] St Croix C. M. Morgan B. J. Wetter T. J. Dempsey J. A. 2000 Fatiguing inspiratory muscle work causes reflex sympathetic activation in humans. J. Physiol.; 529Pt 2:493-504. 11101657 10.1111/j.1469-7793.2000.00493.x PMC2270191

[b49] Stenberg J. Astrand P. O. Ekblom B. Royce J. Saltin B. 1967 Hemodynamic response to work with different muscle groups, sitting and supine. J. Appl. Physiol.; 22:61-70. 6017655 10.1152/jappl.1967.22.1.61

[b50] Vallbo A. B. Hagbarth K. E. Torebjork H. E. Wallin B. G. 1979 Somatosensory, proprioceptive, and sympathetic activity in human peripheral nerves. Physiol. Rev.; 59:919-957. 227005 10.1152/physrev.1979.59.4.919

[b51] Volianitis S. Secher N. H. 2002 Arm blood flow and metabolism during arm and combined arm and leg exercise in humans. J. Physiol.; 544:977-984. 12411540 10.1113/jphysiol.2002.023556 PMC2290626

[b52] Walgenbach S. C. Donald D. E. 1983 Cardiopulmonary reflexes and arterial pressure during rest and exercise in dogs. Am. J. Physiol.; 244:H362-H369. 6829778 10.1152/ajpheart.1983.244.3.H362

[b53] Wells R. Morrissey M. Hughson R. 1986 Internal work and physiological responses during concentric and eccentric cycle ergometry. Eur. J. Appl. Physiol. Occup. Physiol.; 55:295-301. 3732256 10.1007/BF02343802

[b54] Witte K. K. Notarius C. F. Ivanov J. Floras J. S. 2008 Muscle sympathetic nerve activity and ventilation during exercise in subjects with and without chronic heart failure. Can. J. Cardiol.; 24:275-278. 18401467 10.1016/s0828-282x(08)70176-4 PMC2644031

[b55] Yamada Y. Miyajima E. Tochikubo O. Matsukawa T. Ishii M. 1989 Age‐related changes in muscle sympathetic nerve activity in essential hypertension. Hypertension; 13:870-877. 2737724 10.1161/01.hyp.13.6.870

[b56] Young D. B. 2010 Control of cardiac output San Rafael Morgan & Claypool Life Sciences 21634064

